# Nutritional Assessment of Free Meal Programs in San Francisco

**DOI:** 10.5888/pcd10.120301

**Published:** 2013-05-30

**Authors:** Courtney R. Lyles, Soledad Drago-Ferguson, Andrea Lopez, Hilary K. Seligman

**Affiliations:** Author Affiliations: Soledad Drago-Ferguson, Samuels and Associates, Oakland, California, and University of California Berkeley, Department of Public Health Nutrition, Berkeley, California; Andrea Lopez, Hilary K. Seligman, University of California San Francisco Center for Vulnerable Populations, Division of General Internal Medicine at San Francisco General Hospital, San Francisco, California.

## Abstract

Free meals often serve as a primary food source for adults living in poverty, particularly the homeless. We conducted a nutritional analysis of 22 meals from 6 free meal sites in San Francisco to determine macronutrient and micronutrient content. Meals provided too little fiber and too much fat but appropriate levels of cholesterol. They were also below target for potassium, calcium, and vitamins A and E. These findings may inform development of nutritional content standards for free meals, particularly for vulnerable patients who might have, or be at risk of developing, a chronic illness.

## Introduction

Soup kitchens, which serve prepared, generally warm meals and a setting in which to eat, often serve as the primary food source for homeless and marginally housed people (1). Maximizing the nutritional value of meals is often secondary to the soup kitchen’s mission of providing calories, especially given limited financial resources. The nutritional content of soup kitchen menus have been infrequently examined, but the few existing studies suggest that soup kitchens are inconsistently able to provide nutritionally balanced and healthful meals (2–5).

In addition to having unmet nutritional needs (6,7), homeless adults have more difficulty accessing health care than adults who are not homeless and disproportionally suffer from poor health and chronic disease, including diabetes, hypertension, and heart disease (8). Therefore, understanding the nutritional content of free meals is important for this population.

## Methods

From an inventory of existing programs serving free meals in San Francisco (both publicly funded and community-based), we selected those that served at least 1 meal 5 times per week and were open to the community. Ten eligible organizations were identified, and 6 consented to participate. The remaining organizations could not be contacted. Participating organizations represented most free meals served in San Francisco, as sites collectively served an average of 31,000 meals every month; each site served approximately 600 to 18,000 meals per week.

On up to 5 nonconsecutive days during a 10-week period, we observed single meals at each site based on scheduling availability and number of daily meals served (1–5 meals per site, total = 22 meals). We focused on sampling lunches or dinners whenever possible for comparability, as all sites served these daily, and 3 served more than 1 meal daily. We collected recipes and ingredient labels and assessed portion sizes. We then performed an indirect nutritional analysis, calculating averages across sites for the following: calories; percentage of calories from fat; protein; carbohydrates; fiber; cholesterol; vitamins A, C, and E; calcium; iron; sodium; and potassium. We compared values to standards from the Institute of Medicine and the United States Department of Agriculture (USDA) (9,10). We examined nutrient availability using USDA reference standards that represented 2 typical clients: a moderately active man and woman aged 40. Because of the lack of data about the number of meals clients consume daily, we also assessed 2 scenarios for the meal, representing either 40% to 60% of daily intake (which assumes clients are eating approximately 2 meals) or 30% to 36% of daily intake (which assumes clients are eating approximately 3 meals). We considered the meal to be “on target” for macronutrients and micronutrients if it was within the recommended ranges (ie, 40%–60% or 30%–36%). The institutional review board at the University of California, San Francisco approved the study.

## Results

The sample of 22 meals included 17 lunches, 4 breakfasts, and 1 dinner. Meals provided an average of 616 calories, which was inadequate for everyone consuming 2 equicaloric meals daily or men consuming 3 equicaloric meals daily (Table). Meals contained an average of 230 calories from fat, higher than the recommended 20% to 35% of total calories. They also provided inadequate fiber (<30% of daily recommended intake) but appropriate cholesterol (32% of daily recommended intake). Average carbohydrates (56%) and protein (46%) were adequate for those consuming 2 equicaloric meals.

**Table:** Nutritional Analysis Findings

Nutrient	Mean (Range) (n = 22 meals)	% of Reference Standard, 40-Year-Old Man, Moderately Active			% of Reference Standard, 40-Year-Old Woman, Moderately Active		
		100	40–60^a^	30–36^b^	100	40–60^a^	30–36^b^
Calories^c^	616 (438–1,086)	2,600	1,040–1,560	780–936	2,000	800–1,200	600–720
% Calories from fat^d^	36^e^ (15–59)	20–35	20–35	20–35	20–35	20–35	20–35
Protein, g^d^	26 (12–48)	56	22.4–33.6	16.8–20.2	46	18.4–27.6	13.8–16.6
Carbohydrate, g^d^	73 (25–125)	130	52–78	39–47	130	52–78	39–47
Fiber, g^d^	7.1 (2.3–19)	38	15.2–22.8	11.4–13.7	25	10–15	7.5–9.0
Cholesterol, mg^c^	96 (29–481)	300	120–180	90–108	300	120–180	90–108
Vitamin A, µg RAE^d^	194 (5-450)	900	360–540	270–324	700	280–420	210–252
Vitamin C, mg^d^	49 (0-171)	90	36–54	27–32	75	30–45	23–27
Vitamin E, mg AT^d^	1.1 (0.12–3.7)	15	6–9	4.5–5.4	15	6–9	4.5–5.4
Calcium, mg^d^	189 (34–463)	1,000	400–600	300–360	1,000	400–600	300–360
Iron, mg^d^	4.4 (1.1–10.9)	8	3.2–4.8	2.4–2.9	18	7.2–10.8	5.4–6.5
Sodium, mg^d^	944 (381–1,683)	1,500	600–900	450–540	1,500	600–900	450–540
Potassium, mg^d^	626 (100–1,438)	4,500	1,880–2,820	1,410–1,692	4,500	1,880–2,820	1,410–1,692

Abbreviations: RAE, retinol activity equivalent; AT, alpha-tocopherol.

a Assumes soup kitchen meal represents half of daily intake.

b Assumes soup kitchen meal represents one-third of daily intake.

c Reference standard: USDA Dietary Guidelines for Americans: http://www.cnpp.usda.gov/Publications/DietaryGuidelines/2010/PolicyDoc/Appendices.pdf.

d Reference standard: IOM Recommended Daily Allowances (RDA) or Adequate Intake (AI): http://www.iom.edu/Activities/Nutrition/SummaryDRIs/DRI-Tables.aspx.

e Calculated as a percentage of total calories rather than a specific value.

For micronutrients, meals were below target for potassium (13% of daily recommended intake), calcium (19%), vitamin A (22%), and vitamin E (7%). Meals were on target for vitamin C (54%) and, for men only, iron (55%). Sodium content was 63% of daily recommended intake, which was high, particularly for those consuming 3 meals daily.

## Discussion

The nutritional quality of free meals examined in this study was inconsistent in meeting the full range of recommended intake. Our findings were similar to those of other studies, which indicated that soup kitchens provided adequate protein (2–5) but inadequate fiber and micronutrients (obtained predominantly from fruits and vegetables) (2,5). However, conclusions have been conflicting about nutritional standards for calories, fat, sodium, and cholesterol in these settings, often differing by what authors assume a meal represents in an average client’s daily intake (3–5). Standardizing methods would aid comparability and allow tracking of nutritional changes over time.

Monitoring the sodium content of meals is one of the most important nutritional assessments for preventing and managing cardiovascular disease. The average US diet contains 3,400 mg of sodium daily (11), so the 944 mg average per meal we observed is good. However, clients with hypertension or cardiovascular disease would have difficulty achieving the recommended intake for sodium, and clients with diabetes would also struggle to maintain glycemic control with the average carbohydrates per meal. Of note, 3 of the 6 participating sites reported they provided special meals for clients with health conditions (ie, low salt or diabetes-specific), although we evaluated only standard meals in this study.

The economic recession and resulting 24% increase in requests for emergency food has put organizations with limited funding under increasing financial strain (12). Our qualitative experience (not reported) suggests that in areas where soup kitchens are not meeting nutritional needs, it is not from lack of will, but rather lack of money or preparation time, space, or expertise. In addition, soup kitchens’ reliance on food donations often limits availability of healthful alternatives.

Our study has limitations. Most sites in this study reported adhering to either external or internal nutritional standards in creating menus, which may limit the generalizability of these findings. We also examined a small number of meals at each site (most, but not all, of which were lunches), and averaged across sites and meal types. However, results were similar when examining lunches alone. Finally, the conversion of food recipes is an estimate of the nutritional content served, rather than an exact analysis of the food consumed.

Although free meals provided appropriate amounts of several nutrients, which likely represents a concerted effort among soup kitchens to focus on nutritional quality of meals, there is room for improvement. Future work should address nutritional requirements for vulnerable clients with chronic illnesses.
